# Pathway and protein engineering for biosynthesis

**DOI:** 10.1016/j.synbio.2022.06.007

**Published:** 2022-06-22

**Authors:** Yongjin Zhou, Martin Grininger, Hal Alper

**Affiliations:** Dalian Institute of Chemical Physics, Chinese Academy of Sciences, China; Goethe University Frankfurt, Germany; The University of Texas at Austin, USA

Sustainable biosynthesis of chemicals and efforts to create new molecules of interest require efficient enzymes and pathways as well as comprehensive tools and technologies to implement this rewiring. Enzymes are the key components for construction of efficient biosynthetic pathways, **enzyme characterization and engineering** can help to identify key enzymes and regulatory factors for construction of biosynthetic pathways, as well as improve enzyme performance; **Pathway engineering** can help construct biosynthetic pathways and balance metabolic network to improve biosynthetic efficiency; **Tools and technologies** facilitate the engineering of enzymes, pathways, and whole cells. This special issue focusing on “Pathway and Protein Engineering for Biosynthesis” comprises eight review articles and nine original research articles, which highlight and showcase current progress on Pathway and Protein Engineering and their application for biosynthesis.

## 1 Enzyme characterization and engineering

Enzymes are the basic components for biosynthesis. For example, microbial synthesis is considered as a feasible approach for sustainable terpenoid production, which relies on terpenoid synthase as a catalytic enzyme. Ma et al. identified a (−)-bornyl diphosphate synthase from *Blumea balsamifera* and applied it for the biosynthesis of (−)-borneol in yeast [1]. Corpuz et al. reviewed the current progress on protein-protein interface analysis of the non-ribosomal peptide synthetase (NRPS), providing insights for engineering these mega-enzymes [2]. Similarly, Guzman et al. summarized how to use fragment-antigen binding domains as protein crystallization chaperones for structural study of assembly-line polyketide synthases (PKSs), which are of interest to synthesize an unusually broad range of medicinally relevant compounds [3].

Glycosyltransferases (GTs) catalyze the transfer of nucleotide-activated sugars to specific acceptors during biosynthesis of natural product glycosides. He et al. discussed recent progress in the identification and engineering of novel GTs for synthesis of plant natural products [4]. Cytochrome P450 enzymes (CYPs) catalyze a series of C–H and C=C oxygenation reactions for biosynthesis of desired chemicals or pharmaceutical intermediates, a review article by Yan et al. provided a comprehensive overview of CYP function for the C–H and C=C oxygenation reactions and also various strategies for achieving higher selectivity and enzymatic activity [5]. *Vitreoscilla* hemoglobin (VHB) has been widely used to enhance cellular oxygen transfer and metabolite synthesis in fermentation. Zhang et al. optimized the expression cassette of VHB to improve poly-γ-glutamic acid production in *Bacillus licheniformis* [6].

## 2 Pathway engineering

Even with efficient enzymes, biosynthesis pathways should be carefully balanced to enhance net reaction flux. 3-Hydroxypropionic acid (3-HP) is an important platform chemical that can be easily transformed into other valuable compounds such as acrylic acid, acrylamide and 1,3-propanediol. Lai et al. optimized the 3-HP biosynthetic pathway and central metabolism in *E. coli*, which enabled efficient production of 3-HP from syngas-derived acetic acid [7]. Cyanobacteria can utilize CO_2_ to produce a variety of high value-added products through photosynthesis, which involves complex electron transfer process. Fan et al. showcased that enhancing the cellular content of plastoquinone, an important electron carrier, improved the photosynthesis and respiration rate, as well as cellular lipid and protein contents [8]. Ethanol is predominantly used as a renewable ‘drop-in’ transportation fuel and a feedstock for production of other compounds. van Aalst et al. reviewed pathway engineering strategies for improving ethanol yield of anaerobic fermentation of sugars [9]. For heterologous production of spinosad in *Streptomyces albus*, An et al. engineered the polyketide skeleton and precursor supply, which resulted the highest spinosad titer of 70 mg/L in a heterologous *Streptomyces* species [10]. Complex peptide natural products exhibit diverse biological functions and can be served as drug candidates. Wenski et al. overviewed biosynthetic pathways and engineering strategies for two main complex peptides: ribosomally synthesized and post-translationally modified peptides and non-ribosomal peptides [11].

## 3 Tools and technologies

Synthetic biology tools and advanced technologies can accelerate the engineering of the pathways and enzymes in a high throughput manner. Two review articles included in this special issue summarized the recent progresses on technological developments to improve the stress tolerance of microorganisms [12] and engineering of pathways and genomes [13], respectively. Base editing technology has opened a new avenue for genome engineering, however it still suffers from limited availability of editable sites in the target bacterial genome. Chen et al. developed a broad-spectrum DNase-inactive Cpf1 (dCpf1) variant from *Francisella novicida* through directed evolution, which enabled specific C to T mutations at multiple target sites in the *E. coli* genome without compromising cell growth [14]. Construction and balancing of biosynthetic pathways require expression of multiple genes, which is normally realized by different promoters with various strengths. Yan et al. systematically characterized a variety of native promoters and also constructed artificial promoters for metabolic engineering of methylotrophic yeast *Ogataea polymorpha* [15], which will help to construct yeast cell factory for methanol biotransformation. For engineering of *Saccharomyces cerevisiae*, Ambrosio et al. designed and characterized 41 synthetic guide RNA sequences to expand the CRISPR-based genome engineering capabilities, and characterize in high temporal resolution 20 native promoters and 18 terminators [16]. As mentioned above, engineering of methyltrophic yeast can help to establish methanol biotransformation process for chemical biosynthesis, but the complex regulation of methanol metabolism hinders rational engineering. Hou et al. carried out comparative proteomics analysis of *Pichia pastoris* cultivated in glucose and methanol, which identified several genes that play important roles in methanol utilization [17].

We thank all contributing authors for making this special issue on “Pathway and protein engineering for biosynthesis” possible, and also the reviewers for their time and constructive comments throughout the reviewing process to improve the manuscripts. We hope that readers find these articles interesting and inspiring to their own research.
